# Influence of the Socio-Economic Context on Self-Reported Gingival Bleeding in Individuals of Ethnic Minority Groups: A Multilevel Analysis

**DOI:** 10.5539/gjhs.v8n2p1

**Published:** 2015-06-01

**Authors:** Carlos M. Ardila, Annie Marcela Vivares-Builes, Andrés A. Agudelo-Suárez

**Affiliations:** 1 Biomedical Stomatology Research Group, Department of Periodontology, School of Dentistry, Universidad de Antioquia U de A, Medellín, Colombia; 2 Basic Studies Department, School of Dentistry, Universidad de Antioquia, Medellín, Colombia; 3 Research Department, School of Dentistry, Universidad de Antioquia, Medellín, Colombia

**Keywords:** ethnic groups, gingival hemorrhage, healthcare disparities, multilevel analyses, self report

## Abstract

**Introduction:**

To evaluate the influence of the socio-economic context on self-reported gingival bleeding (SRGB) in individuals of ethnic minority groups (IEG).

**Methods:**

Data from the 2007 National Public Health Survey in Colombia were collected. A multiple-stage stratified sampling was used. Data from 34.843 subjects were collected through interviews. The influence of socio-economic factors on SRGB in IEG was investigated with logistic and multilevel regression analyses.

**Results:**

Out of 34.843 subjects studied, a total of 6.440 individuals were members of ethnic minority groups. SRGB was observed in approximately 5% of IEG. There was a significant difference between IEG and subjects of the rest of the sample (28.403 subjects) regarding SRGB, elementary and high school education, Gross Domestic Product (GDP), Human Development Index (HDI) and Unmet Basic Needs Index (UBNI) disfavouring IEG (P<0.05). The logistic model showed that SRGB was associated with IEG (P<0.001). This association persisted after controlling for confounders. A total of 33 Colombian states (level 2) and 6.440 members (level 1) of ethnic minority groups were included in the multilevel analisys; this model showed that the variance on SRGB was statistically significant at level 1 and 2. However, the variation at IEG level (35%) was smaller than the variation between states (65%) in the multilevel multivariate model.

**Conclusions:**

SRGB was higher in IEG. Also, GDP, HDI and UBNI were unfavourable factors in the members of ethnic minority groups. Considering these detriment factors and the higher variation between states, this study suggests that socio-economic context affects significantly SRGB in IEG.

## 1. Introduction

Gingival bleeding indicates the manifestation of an inflammatory lesion that also is considered as the most perceptive sign of initial gingival inflammation. Gingival bleeding appears together with other expressions of periodontal diseases like gingival swelling, gingival recession, and tooth mobility (Armitage, 1999). The blood from gingival bleeding may be noticed on the affected person’s breath and is observed frequently during tooth brushing, becoming a complaint among patients with gingivitis and periodontitis (Azodo et al., 2011). Gingival bleeding is the most predominant sign of periodontitis around the world, being a relevant oral disease providing to the worldwide assessment of chronic sickness, representing a significant public health problem (Petersen & Ogawa, 2012).

Self-report is commonly utilized to estimate the occurrence of several medical diseases and is an alternative to clinical periodontal evaluation. Researchers have contemplated self-reported gingival bleeding (SRGB) a valuable manner for examination the gingival health of people in clinical and oral health promotion in order to improve periodontal health. Also, several studies showed that self-reported periodontal diseases were corroborated by clinical evaluation (Airila-Månsson et al., 2007; Azodo et al., 2011), specifically, as pointed out by Gilbert and Litaker (2007), self-rated gingival health and the existence of a loose tooth were the only periodontal parameters that were associated significantly with clinically determined periodontal status in multivariable regression models. They indicated also that significant minority disparities in the validity of self-report were manifested.

Oral health disparities in ethnic minority groups have been extensively recognized (Kim et al., 2012; Craig et al., 2003); these inequities were ascribed to a complicated network of emotional, social and basic aspects, such as use or admission to medical services, nutrition and oral hygiene (Sabbah et al., 2009). A disparity in periodontitis frequency and magnitude has been described for African-Americans in comparison to other American groups. Also, disparities in risk factors for periodontitis were informed for African-American minorities in connection with augmented illness occurrence and gravity. Nevertheless, it is not clear if these dissimilarities are due to other risk variables including genetic, socio-economic condition, admission to oral health assistance, customs or other ecological elements (Craig et al., 2003). Therefore, whether risk factors for disorders advance differ in members of ethnic minorities, subsequently incorrect treatments could be used in these groups (Craig et al., 2003).

The causes of oral illnesses are elucidated by elements at the subject level. Nonetheless, subject proximal features are insufficient elucidations of the frequency and dissemination among communities (Koltermann et al., 2011). Therefore, multilevel analysis has simplified the evaluation of the socio-economic impact and their exchanges with dental status (Roncalli et al., 2014). It permits the integration of socio-economic factors on to the regression analysis to avoid a remaining confusing result of ignoring contextual features, overestimation of the relationship, and underestimation of the standard error (Goldstein et al., 2002). Besides, there is a wide gap in the epidemiological knowledge considering the common influence that the community context has on the occurrence of SRGB.

To the authors´ knowledge, no studies have analyzed the social context impact in explaining SRGB related inequalities. Consequently, the objective of the current study was to evaluate the influence of the socio-economic context on SRGB in individuals of Colombian ethnic groups using a multilevel approach.

## 2. Method

### 2.1 Sources of Data

Data from the 2007 National Public Health Survey in Colombia (ENSP, 2007) were collected (Ministry of Social Protection National Public Health Survey, 2007). The Colombian Ministry of Social Protection (in 32 states and 1 Capital District in the country, as well as the 32 state capitals) conducted this household-based survey. A multiple-stage stratified sampling was used: first-stage units were municipalities or the combination of one or more in the case of small towns; second-stage units were blocks in case of urban regions and in census tracts in rural areas; and the third-stage units were family households. Within each household, one adult was selected to complete the survey. Geographical areas containing different ethnic minority groups such as Indigenous, Afro-descendants, Raizal, Palenquero and Rom or Romani, allowing the evaluation of ethnic differences; these data represent the entire residents.

The present investigation integrated persons aged 18 and elder and excluded the edentulous and those who did not complete the survey; thus, the information of 34.843 participants of the survey population was utilized. Data were obtained through interviews at home between January 2007 and April 2008. For additional information, see the publication of the Ministry of Social Protection, Republic of Colombia (Ministry of Social Protection National Public Health Survey, 2007). Socio-economic referents were acquired from diverse databases from National Demographic Census, effectuated by the Colombian National Administrative Department of Statistics (DANE). Data related to health system indicators were gathered from the health information systems of the Colombian Ministry of Health.

### 2.2 Ethics

This investigation was established on secondary data acquired from numerous publicly accessible data sets, consequently did not require ethical approval; however ethical approval proceeded of the ENSP-2007 conducted by the Ministry of Social Protection of the Colombian government (Reference: 519-2008).

### 2.3 Statistical Analysis

The Kolmogorov-Smirnov test checked the normal distribution of quantitative variables. Qualitative variables were contrasted with the *X*^2^ test, and independent t-test was used to determine the differences between groups (individuals of ethnic groups and the subjects from the rest of the population). Crude and multivariate logistic regression models were performed to define the discriminative significance of SRGB for individuals of ethnic minority groups (IEG). Confounder variables included individual factors known: age (in years), gender (male/female) and education level (without studies or elementary school; high-school; and college education).

The odds ratios (ORs) were controlled for the above-described covariable in the logistic regression models. The ORs and related 95% confidence intervals (CI) were estimated for each variable. P values <0.05 were considered statistically significant. All analyses were performed using statistical software (SPSS version 18.0; SPSS, Chicago, IL).

The influence of different factors on the outcome (SRGB) was investigated with multilevel regression analyses. A two-level random intercept regression model was constructed: IEG at level 1 and Colombian states at level 2 (Thirty-two states and one Capital District). A variance components model (null model) was constructed using SRGB as the dependent variable but without introducing explanatory variables. The null model was performed to estimate the complete variability of SRGB and to apply it to IEG and Colombian states levels. Subsequently, a sequence of explicatory variables was included (multivariate model). This additional phase permitted the inspection of the connection between each co-variable and the dependent variable.

The explanatory variables at level 1 were: gender (male/female), age (in years), and education level (college education=yes/no).

The explanatory variables at level 2 were: Gross Domestic Product (GDP=aggregate measure of production equal to the sum of the gross values added of all resident institutional units engaged in production by region or state), Human Development Index (HDI=geometric average of the indices income, education and longevity, with equal weights) and Unmet Basic Needs Index (UBNI=the access to a dwelling which guarantees a minimum housing standard [construction materials and overcrowding]; the access to basic sanitary facilities in the dwelling; the access to basic education, the economic capacity to reach minimum consumption levels).

P values <0.05 were contemplated statistically significant. A statistical package designed specifically for multilevel modeling was used (Multilevel Models Project Institute of Education, MLwiN, version 2.16, London, U.K)

## 3. Results

As shown in Table 1, the female was the most common gender while college education was the lowest education level observed. The average age of the subjects was approximately 39 years and approximately 18% of the individuals were members of ethnic minority groups. SRGB was observed in almost 4% of the sample and in 5.1% of IEG.

**Table 1:** Socio-demographic characteristics and self-reported gingival bleeding in 34.843 subjects studied

Parameter	Value
Age (years±standard deviation)	39.4±14
Gender	
% Female	56.8
% Male	43.2
Education level	
% Without studies or elementary school	45.6
% High-school	46.2
% College	8.2
Number and % of individuals of an ethnic group	6.440 (18.5)
Number and % of subjects of the rest of the population	28.403 (81.5)
Number and % of self-reported gingival bleeding	1.318 (3.8)

The percentage of SRGB and contextual variables at each Colombian state are listed in Table 2. SRGB scores range from 1.4% to 7.7%. GDP, HDI and UBNI scores present the best performance in the Capital District.

**Table 2:** Percentage of self-reported gingival bleeding and contextual variables at 32 Colombian states and 1 Capital district^*^

State	% Self reported gingival bleeding	GDP	HDI	UBNI
Amazonas	6.3	0.66	0.76	22.8
Antioquia	3.45	0.73	0.78	17.1
Arauca	3.2	0.66	0.76	22.8
Atlántico	2.75	0.67	0.79	16.1
Bogotá*	4.4	0.75	0.83	6.6
Bolivar	7.3	0.66	0.77	30
Boyaca	4.45	0.67	0.76	19.3
Caldas	2.35	0.68	0.77	13.7
Caquetá	4.05	0.61	0.73	26.9
Casanare	1.75	0.66	0.76	22.8
Cauca	5.15	0.61	0.76	23.3
César	2.15	0.67	0.76	35.7
Chocó	4.55	0.54	0.67	67.1
Córdoba	3.6	0.65	0.75	35.8
Cundinamarca	3.7	0.7	0.79	17.8
Guainía	3.8	0.66	0.76	22.8
Guaviare	2.9	0.66	0.76	22.8
Huila	2.75	0.67	0.77	21.7
Guajira	7.15	0.71	0.78	37.5
Magdalena	3.2	0.59	0.74	39.6
Meta	6.4	0.71	0.76	18.6
Nariño	4.15	0.58	0.72	28.7
Norte de santander	1.4	0.6	0.74	22.8
Putumayo	2.75	0.66	0.76	22.8
Quindio	2.85	0.63	0.76	11.8
Risaralda	2.65	0.65	0.77	13.2
San Andres	5.5	0.66	0.76	22.8
Santander	2.25	0.74	0.8	13.2
Sucre	4.45	0.55	0.73	42.4
Tolima	4.25	0.67	0.76	22.5
Valle del Cauca	3.3	0.72	0.79	12.6
Vaupes	3.8	0.66	0.76	22.8
Vichada	7.7	0.65	0.73	21.9
Total Colombia	3.8	0.7	0.78	19.3

GDP= Gross Domestic Product;

HDI= Human Development Index;

UBNI= Unmet Basic Needs Index.

Table 3 depicts the socio-demographic characteristics and SRGB of the members of ethnic groups or not. There was a significant difference between IEG (6.440 subjects) and the rest of the subjects of the sample (28.403 individuals), regarding SRGB, elementary and high school education, GDP, HDI and UBN scores, disfavouring IEG (P<0.05). In IEG, GDP and HDI scores are below the national average while UBN is above.

**Table 3:** Comparison of the socio-demographic characteristics and self-reported gingival bleeding of the persons of an ethnic group (n=6.440) and subjects of the rest of the sample (n=28.403)

Parameter	Ethnic group	Rest of the sample	P value
Age (years± standard deviation)	39.5±14	39.1±13	NS
Gender			
% Female	51.5	58	NS
% Male	48.5	42	NS
Education level			
% Without studies or elementary school	48.9	44.8	<0.05
% High-school	43.4	46.7	<0.05
% College	7.7	8.5	NS
% Self-reported gingival bleeding	5.1	3.4	<0.05
GDP	0.63	0.70	<0.05
HDI	0.75	0.78	<0.05
UBNI	32	20.7	<0.05

NS= not statistically significant;

GDP= Gross Domestic Product;

HDI= Human Development Index;

UBNI= Unmet Basic Needs Index.

In the unadjusted logistic regression model, SRGB was associated with IEG (P<0.001). This significant association persisted after controlling for probable confounders (Table 4). It is fundamental to stand out that the variable age was statistically significant in the model (OR=1.015, 95% CI = 1.01-1.02, P<0.001).

**Table 4:** Multivariable regression analysis on self-reported gingival bleeding in members of ethnic groups (6.440 individuals)

Variable	OR Self-reported gingival bleeding	(95% CI)	*P* Value
Ethnic group	1.51^*^	(1.32-1.71)	<0.001
	1.5^**^	(1.31-1.72)	<0.001

* Unadjusted;

** Adjusted for age, gender and education level.

A total of 32 Colombian states and 1 Capital District and 6.440 IEG were included in the multilevel analysis. The multilevel analysis showed that the variance on SRGB was statistically significant at level 1 and 2 (Table 5). The variation at IEG level (36%) was smaller than the variation between states (64%).

**Table 5:** Multilevel logistic analysis valuing the influence of individual and contextual characteristics to the variability on self-reported gingival bleeding in 6.440 individuals and 33 states

Intercept	Self-reported gingival bleeding

	Crude model β±SE^*^	Multivariate model β±SE
Variance	3.306±0.083	2.765±0.145
State (Level 2)	0.105±0.045^*^	0.099±0.044^*^
Ethnic (Level 1)	0.058±0.025^*^	0.053±0.026^*^
Total variance	0.163	0.152

SE=Standard error;

* P<0.05.

Regression estimates and significance testing for all ethnic and contextual variables were also performed. Table 6 shows the regression estimates for covariates. Newly, the multilevel analysis showed that the variance on SRGB was statistically significant at level 1 and 2 and the variation at IEG level (35%) was smaller than the variation between states (65%). Besides, the multilevel analysis associated SRGB with age (P=0.006). As presented above, this result was also corroborated by the logistic regression analyses. No other ethnic or contextual variables were significant in the model.

**Table 6:** Multilevel logistic model evaluating the significance of ethnic and state features explicating the variability on self-reported gingival bleeding in 6.440 individuals and 33 states

Parameters	Self-reported gingival bleeding (β±SE)	*P* value
Ethnic (Level 1)		
Age	0.015±0.002	0.006
Gender	0.075±0.085	NS
Education level	0.090±0.068	NS

State (Level 2)		
GDP	2.139±2.074	NS
HDI	0.087±5.572	NS
UBNI	0.010±0.010	NS

±Standard error;

NS= not statistically significant;

GDP= Gross Domestic Product;

HDI= Human Development Index;

UBNI= Unmet Basic Needs Index.

## 4. Discussion

This was the first research that investigates the social context impact in explaining SRGB related inequalities. The implemented hierarchical model contemplated factors from the contextual level as being mediators to the entire complex of individual determination.

SRGB could be one of the best consistent markers of the complete estimation of oral status being confirmed by clinical examinations (Airila-Månsson et al., 2007). In this study, 5.1% of members of ethnic minority groups reported gingival bleeding, prevalence similar to those described by other researchers. They also observed self-evaluation of bleeding as an advantageous approach for examining gum status of communities (Mariño et al., 2008).

This research shows SRGB, elementary and high school education, GDP, HDI and UBN scores, disfavouring IEG (P <0.05). In line, higher severe periodontitis frequency was observed in African-American minorities, suggesting that contextual characteristics may have a superior effect on risk factors related with disorder occurrence and advancement in these ethnic groups (Craig et al., 2003).

In the current study, the adjusted logistic regression model for SRGB was associated with IEG, and the variable age was statistically significant. Increasing age as a possible factor associated with an increase in periodontal diseases in members of ethnic groups has been documented previously (Craig et al., 2003; Filho et al., 2014). This highlights the progress of dental health complications; it emphasizes also the significance of prevention and prompt intermediation for ethnic minority groups and socioeconomic subgroups in averting oral health inequalities in old age (Kim et al., 2012).

Although the variable smoking status was not contemplated in the present study, smokers were more likely to describe their periodontal tissues as unhealthy, whereas self-reports of healthy versus unhealthy gums did not differ between non-smokers (Özçaka et al., 2014). Additionally, smoking had a robust inhibition result on gum bleeding and the result was greatest in heavier smokers and lowest in actual smokers (Dietrich et al., 2004). However, Colombia has a low prevalence of smoking in Latin America (Rivera-Andrade & Luna, 2014) and this fact could not affect the frequency on SRGB in the present study.

This investigation studied SRGB beyond the conventional manner of observing the risk factors for gum bleeding, valuing the connection between the environment of the population where the members of ethnic groups were inhabiting, after adjusting for subject and contextual characteristics. The multilevel analysis showed that the variance on SRGB was statistically significant at level 1 and 2 in the null model. After adjustment for individual and community features, state level variance still continued significant. This result suggests that contextual factors affect SRGB. Consequently, there could be benefits to emphasis on community health strategies not simply for subjects but likewise for high self-reported minorities. A complete community methodology and a pointed approach could achieve successful policies for overcoming gingival bleeding inequality.

The results in relation to the contextual influence on SRGB are not definitely elucidated since there are not equivalent investigations. However, considering other publications with diverse objectives, the contextual consequences were either at national, region, state or city levels. Thus, contextual consequences were also observed for dental treatment needs (Roncalli et al., 2014), dental status (Sander et al., 2008), dental pain (Santiago et al., 2013) and dental caries (Aida et al., 2008).

The present study has several strengths. First, nationwide demonstrative information with good personal and domestic response rates were used. Second, the sample magnitude likewise permits the handling of consistent populace estimations with narrow confidence intervals, and third, the use of multilevel exploration in this investigation made it probable to observe the results of the socio-economic elements on SRGB. However, a limitation was the utilization of results acquired from a cross-sectional investigation, which prevents a classic exploration of causality. Besides, subject knowledge and evidence on the population level factors disturbing gingival bleeding were restricted. More affordable features originated from qualitative investigation representing social context could have diminished the unsolved contextual-level variability (Ocampo, 2003).

In conclusion, SRGB was higher in members of ethnic minority groups. Also, GDP, HDI and UBNI were unfavourable factors in the members of ethnic minority groups. Considering these detriment factors and the higher variation between states, this study suggests that socio-economic context affects significantly SRGB in IEG. More reasonable characteristics of socio-economic context are needed to explain the forms by which context modifies SRGB.
